# Age‐dependent effect of thrombolytics on likelihood of National Institutes of Health Stroke Scale improvement in minor strokes

**DOI:** 10.1002/ibra.70008

**Published:** 2025-12-01

**Authors:** Tarun Thomas, Michael J. Dubinski, Omnea Elgendy, Sonali Vij, Lucas P. Garfinkel, Karan Patel, Jesse M. Thon, Pratit D. Patel, Hamza A. Shaikh, Jane Khalife, Hermann C. Schumacher, Khalid A. Hanafy, Tudor G. Jovin, Manisha Koneru

**Affiliations:** ^1^ Cooper Neurological Institute Cooper University Health Care Camden New Jersey USA; ^2^ Department of Neuroscience Cooper Medical School of Rowan University Camden New Jersey USA

**Keywords:** acute ischemic stroke, alteplase, tenecteplase, thrombolysis

## Abstract

The appropriate acute treatment strategy for minor ischemic stroke, defined as National Institutes of Health Stroke Scale scores ≤5, is not as well‐defined. Prior studies have demonstrated mixed results regarding the effects of neurovascular interventions on minor stroke patients for optimizing the chances of symptomatic improvement. We performed a retrospective single‐center study across 6 years to determine the association between thrombolytics and the likelihood of clinical symptom improvement by 24 h in minor stroke patients across ages. Margin plots were derived from multivariable regression analyses. Of 1172 minor stroke patients, in patients <70 years of age, there was greater than 50% likelihood of improvement with any type of thrombolytic administration. When substratifying by type of thrombolytic, there is greater than 50% odds of improvement in patients <80 years of age treated with alteplase and <70 years of age treated with tenecteplase. Thus, the association between age and likelihood of benefit after thrombolytic treatment in minor stroke patients highlights particular minor stroke subpopulations, particularly younger patients, who may benefit from thrombolytic treatment.

## Graphical abstract

There is mixed evidence regarding thrombolytics for minor stroke (NIHSS ≤ 5) patients, particularly across different subpopulations. There is an association between age and treatment response to thrombolytic therapy; there is an age‐dependent effect of degree of NIHSS improvement in response to thrombolytic treatment, particularly with tenecteplase.

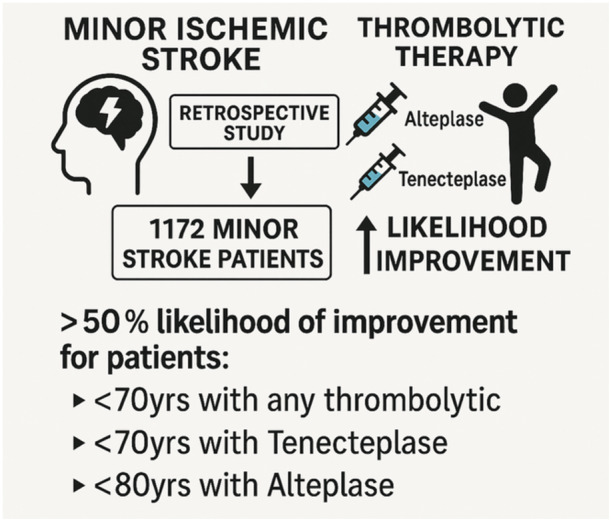

## 1 INTRODUCTION

Acute ischemic stroke is one of the leading causes of disability and cardiovascular mortality worldwide.^1^ The treatment of acute ischemic stroke with intravenous thrombolytics (IVT) and endovascular thrombectomy (EVT) has been shown to have rapid therapeutic effects, decreasing mortality and improving odds of functional recovery.^2^, ^3^, ^4^, ^5^, ^6^ Although the treatment benefit of thrombolytics and EVT in large vessel occlusion strokes, including in patients presenting in the late windows and with large core volumes, has been explored, the optimal treatment strategy for minor strokes is not as well‐defined.^7^, ^8^, ^9^, ^10^, ^11^, ^12^, ^13^, ^14^, ^15^, ^16^, ^17^, ^18^, ^19^, ^20^

Minor strokes, defined as National Institutes of Health (NIHSS) stroke score of 5 or less, often present with mild symptoms.^21^, ^22^ In conjunction to a higher risk of further acute deterioration, there also needs to be a balance between the clinical benefits of an intervention and the risk of harm, including from post‐interventional symptomatic intracranial hemorrhage.^12^, ^23^, ^24^, ^25^, ^26^, ^27^, ^28^ Historically, IVT has been used to treat minor strokes, including alteplase (tPA) and, more recently, tenecteplase (TNK).^29^ However, the evidence for thrombolytics in minor stroke patients has been heterogeneous. Moreover, there may be an age‐dependent effect of the treatment benefit, especially as older patients may have a risk of hemorrhage and harm from thrombolytics.^30^ This study aims to explore how the likelihood of NIHSS improvement within 24 h in minor stroke patients after thrombolytic treatment may vary across different age groups.

## 2 METHODS

Deidentified data may be made available upon reasonable request to the corresponding author. This retrospective study was approved by the institutional review board with waiver of informed consent. Analysis was reported in accordance with Strengthening the Reporting of Observational Studies in Epidemiology guidelines.

### 2.1 Patient population

A retrospective registry of consecutive acute stroke patients presenting to Cooper University Health Care, a comprehensive stroke center, between January 2017 and September 2023, was reviewed. Patients were included for analysis if the patient had a NIHSS ≤5 upon presentation. Patients were further included for analysis if NIHSS at 24 h after presentation was available, and if the patient's age was available. Patients treated with mechanical thrombectomy were excluded to analyze only patients receiving pharmacological reperfusion therapy (IVT+) or no reperfusion therapy (IVT−).

### 2.2 Data collection

Data collection included the following demographics: age, sex, premorbid modified Rankin Score (mRS), past medical history (prior stroke, hypertension, coronary artery disease, atrial fibrillation, diabetes mellitus, and hyperlipidemia), tobacco use, and prior antiplatelet/anticoagulant use. Stroke characteristics were collected, including: admission NIHSS score, type of thrombolytic administered (i.e., tPA or TNK), NIHSS at 24‐h follow‐up, discharge mRS, and 90‐day mRS.

### 2.3 Statistical analysis

The primary outcome was improvement in NIHSS score by at least 1 point by 24‐h follow‐up. Continuous measures were summarized using medians and interquartile ranges (IQR), and categorical variables were summarized with frequencies. The cohort was analyzed based on thrombolytic administration (IVT+ and IVT−), and further sensitivity analyses were conducted by stratifying the IVT+ cohort by the type of thrombolytic that was administered (i.e., tPA vs*.* TNK). Multivariable age‐adjusted logistic regressions were performed to assess the interaction between age and thrombolytic administration on the odds of NIHSS improvement. Significance of the effect of the interaction term (primary analysis: age and IVT administration or sensitivity analysis: age and type of IVT) and main covariates (i.e., primary analysis: age and IVT administration or sensitivity analysis: age and type of IVT) were assessed using the likelihood ratio test. Margin plots were derived to show the predicted probability of NIHSS improvement across age. Absolute benefit increase (ABI) was calculated by comparing the following pairs of cohorts: IVT+ and IVT−, TNK and IVT−, and tPA and IVT−. Missing data was minimal and not imputed. Statistical analyses were performed using JMP v. 18 (SAS Institute Inc.). *p* < 0.5 was considered statistically significant.

## 3 RESULTS

Of 1172 minor stroke patients, the average age was 67 years (IQR 58–76), and 55.9% (655/1172) were female (Table 1). The most common comorbid condition was hypertension (80.8%) (Table 1). Thrombolytics (IVT+) were administered in 9.7% of patients. Of patients treated with IVT, 72.8% were treated with tPA, and 27.2% were treated with TNK (Table 1).

**Table 1:** Demographic data for minor stroke patients.

Variable	All (*n* = 1172)	IVT− (*n* = 1058)	IVT+ (*n* = 114)
Age (years), median (IQR)	67 (58–76)	67 (58–76)	64 (56–74)
Female, no. (%)	655 (55.9%)	586 (55.4%)	69 (60.5%)
Premorbid mRS, median (IQR)	0 (0–1), *n* = 1114	0 (0‐1), *n* = 1005	0 (0–0), *n* = 109
Past Medical History, no. (%)			
Prior Stroke	269 (23.1%), *n* = 1165	252 (24.0%), *n* = 1051	18 (15.8%)
Hypertension	941 (80.8%), *n* = 1165	856 (81.4%), *n* = 1051	85 (74.6%)
Coronary Artery Disease	267 (22.9%), *n* = 1164	239 (22.8%), *n* = 1050	28 (24.6%)
Atrial Fibrillation	207 (17.8%), *n* = 1164	191 (18.2%), *n* = 1050	16 (14.0%)
Diabetes Mellitus	448 (38.5%), *n* = 1164	419 (39.9%), *n* = 1050	29 (25.4%)
Hyperlipidemia	717 (61.7%), *n* = 1163	661 (63.0%), *n* = 1049	56 (49.1%)
Tobacco Use, no. (%)	651 (56.0%), *n* = 1163	600 (57.2%), *n* = 1049	51 (44.7%)
Antiplatelet/Anticoagulation, no. (%)	537 (45.8%), *n* = 1172	491 (46.4%)	46 (40.4%)
Admission NIHSS, median (IQR)	2 (1–3)	2 (1–3)	4 (2–5)
Thrombolytic Administered, no. (%)	‐	‐	
tPA	‐	‐	83 (72.8%)
TNK	‐	‐	31 (27.2%)
NIHSS at 24 h, median (IQR)	1 (0–3)	1 (0–3)	2 (0–4)
Improvement in NIHSS, no. (%)	365 (31.1%)	303 (28.6%)	62 (54.4%)
Discharge mRS, median (IQR)	3 (1–4), *n* = 1101	3 (1–4), *n* = 991	1 (1–4), *n* = 110
Discharge mRS 0–2, no. (%)	532 (48.3%), *n* = 1101	466 (47.0%), *n* = 991	66 (60.0%), *n* = 110
In‐Hospital Mortality, no. (%)	23 (2.1%), *n* = 1101	20 (2.0%), *n* = 991	3 (2.7%), *n* = 110
90‐day mRS, median (IQR)	1 (1–3), *n* = 865	2 (1–3), *n* = 782	1 (0–2), *n* = 83
90‐day mRS 0–2, no. (%)	591 (68.3%), *n* = 865	525 (67.1%), *n* = 782	66 (79.5%), *n* = 83
90‐day Mortality, no. (%)	85 (9.8%), *n* = 867	79 (10.1%), *n* = 784	6 (7.2%), *n* = 83

Abbreviations: IQR, interquartile range; IVT, intravenous thrombolytics; mRS, modified Rankin Scale; NIHSS, National Institutes of Health Stroke Scale; tPA, alteplase; TNK, tenecteplase.

Median admission NIHSS in IVT+ patients was 4 (IQR 2–5), while median admission NIHSS in IVT− patients was 2 (IQR 1–3) (Table 1). Median NIHSS at 24 h in IVT+ patients was 2 (IQR 0–4) and in IVT− patients was 1 (IQR 0–3) (Table 1). More than half of patients treated with IVT improved in NIHSS score by 24 h (54.4%), while only approximately one‐fourth of patients not treated with IVT had improvement in NIHSS score by 24 h (28.6%) (Table 1).

### 3.1 Age and thrombolytic administration

**Figure 1:**
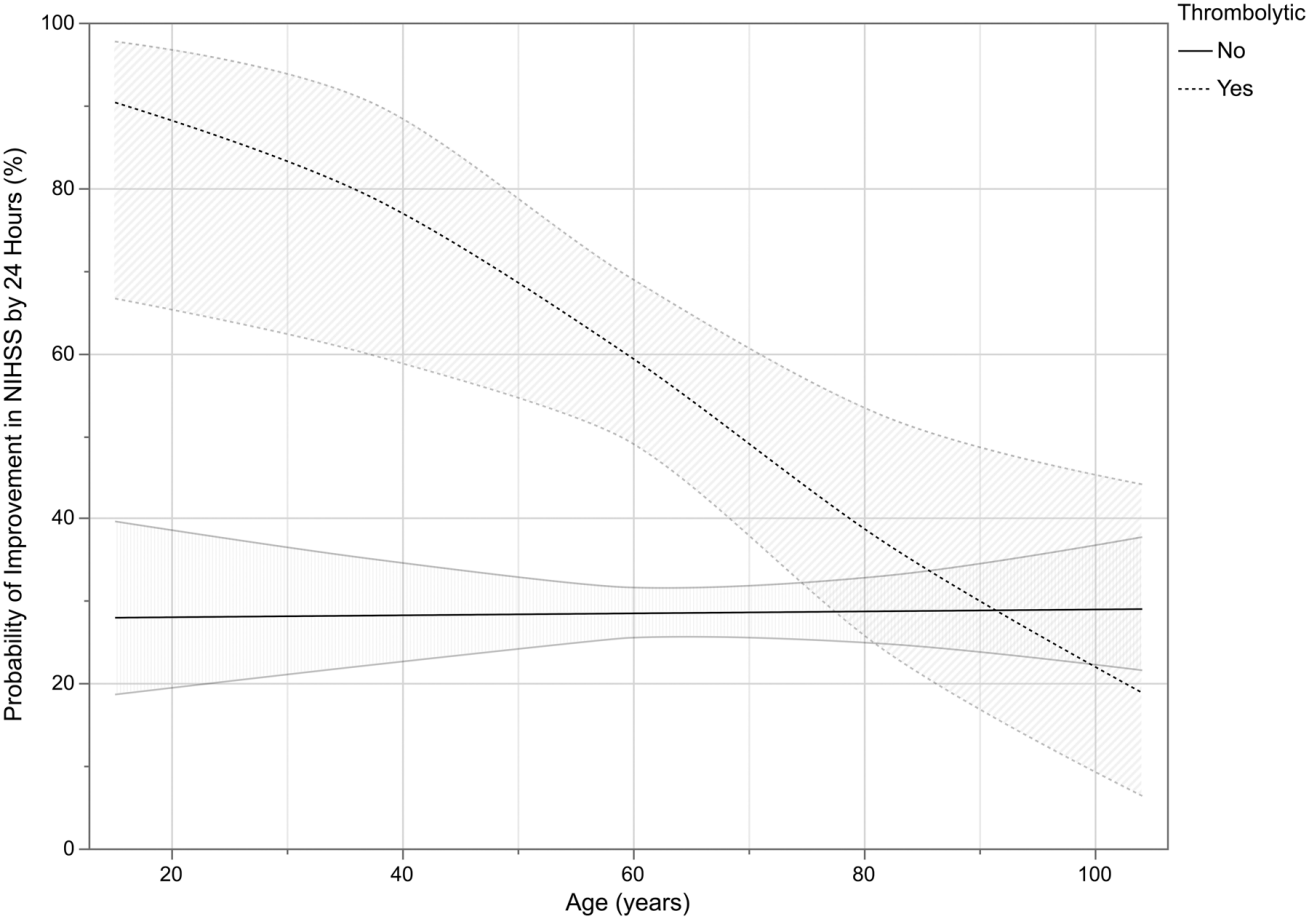
Margin plots showing predicted probability with 95% confidence interval of NIHSS improvement by 24 h after either receiving thrombolytic treatment or no intervention in minor strokes. NIHSS, National Institutes of Health Stroke Scale.

In multivariable regression analysis for improvement in NIHSS by 24 h, age (*p* = 0.01), IVT administration (*p* < 0.001), and the interaction effect between age and IVT administration (*p* = 0.01) were significant (Figure 1). In a margin plot showing predicted probability of improvement of NIHSS by 24 h across ages when stratified between IVT+ and IVT− cohorts, there is greater than 50% likelihood of NIHSS improvement for patients <70 years when treated with IVT (Table 2; Figure 1). The ABI for IVT treatment compared to no IVT treatment is greater than 40% in patients <50 years (Figure 1; Table 2).

**Table 2:** Predicted probability of improvement in NIHSS and absolute benefit increase of thrombolytic administration in minor strokes.

Age (years)	Predicted probability of NIHSS improvement		Absolute benefit increase (IVT+) ‐ (IVT−)
	IVT−	IVT+	
20–29	28.10%	88.47%	60.37%
30–39	28.21%	83.51%	55.30%
40–49	28.33%	76.97%	48.64%
50–59	28.45%	68.81%	40.36%
60–69	28.56%	59.28%	30.72%
70–79	28.68%	48.99%	20.31%
80–89	28.80%	38.80%	10.00%
>90	28.92%	29.49%	0.57%

Abbreviations: IVT, intravenous thrombolytics; NIHSS, National Institutes of Health Stroke Scale.

### 3.2 Age and type of thrombolytic administration

**Figure 2:**
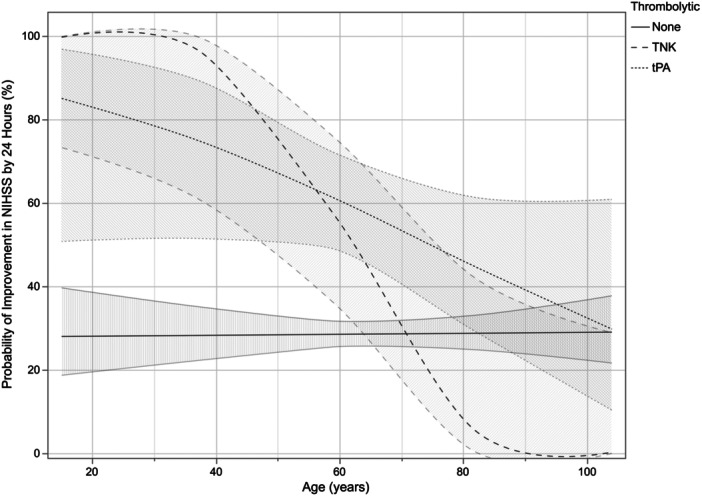
Margin plots showing predicted probability with 95% confidence interval of NIHSS improvement by 24 h after either receiving TNK, tPA, or no intervention in minor strokes. Abbreviations: NIHSS, National Institutes of Health Stroke Scale; TNK, tenecteplase; tPA, alteplase.

In multivariable regression analysis for improvement in NIHSS by 24 h, age (*p* < 0.001), type of IVT administered (*p* = 0.002), and the interaction effect between age and type of IVT (*p* < 0.001) were significant (Figure 2). In a margin plot showing predicted probability of improvement of NIHSS by 24 h across ages when stratifying for type of IVT administration (i.e., tPA v. TNK v. None), there is greater than 50% likelihood of NIHSS improvement in patients <80 years when treated with tPA and patients <70 years when treated with TNK (Table 3; Figure 2). There is also a greater than 80% probability of NIHSS improvement with TNK administration for patients <50 years, while the probability of NIHSS improvement with tPA administration only exceeds this threshold for patients <20 years (Figure 2; Table 3). The ABI for TNK administration compared to no treatment is greater than 50% in patients <50 years; the ABI tPA administration compared to no treatment is greater than 50% in patients <30 years (Figure 2; Table 3). However, in older patients, the ABI for either tPA or TNK reduces (Table 3).

**Table 3:** Predicted probability of improvement in NIHSS and absolute benefit stratified by type of thrombolytic administration in minor strokes.

Age (years)	Predicted probability of NIHSS improvement			Absolute benefit increase compared to no thrombolytic	
	None	tPA	TNK	tPA	TNK
20–29	28.10%	83.21%	99.76%	55.11%	71.66%
30–39	28.21%	78.72%	98.69%	50.51%	70.48%
40–49	28.33%	73.41%	95.01%	45.08%	66.68%
50–59	28.45%	67.33%	82.72%	38.88%	54.27%
60–69	28.56%	60.60%	54.64%	32.04%	26.08%
70–79	28.68%	53.45%	23.27%	24.77%	−5.41%
80–89	28.80%	46.15%	7.09%	17.35%	−21.71%
>90	28.92%	39.02%	1.88%	10.10%	−27.04%

Abbreviations: NIHSS, National Institutes of Health Stroke Scale; tPA, alteplase; TNK, tenecteplase.

## 4 DISCUSSION

We demonstrate an age‐dependent association between thrombolytic administration and the likelihood of NIHSS improvement in minor stroke patients. We further demonstrate that this age‐dependent effect is different between tPA and TNK. In general, any type of IVT administration was associated with more than 50% odds of improvement in patients less than 70 years of age, and the ABI for IVT is greater than 40% in patients less than 50 years of age. When stratified by IVT type, it was found that TNK administered in patients under 70 and tPA administered in patients under 80 was found to have more than 50% likelihood of improvement.

A major proportion of the acute ischemic stroke population constitutes minor stroke patients. Due to the nature of the NIHSS scale, a low score does not necessarily mean the patient does not have debilitating symptoms, as patients with minor stroke often experience focal deficits, loss of concentration, mental fatigability, emotional lability, and other symptoms that drastically decrease quality of life.^31^, ^32^, ^33^ Furthermore, it has been shown that the risk of recurrent stroke following minor stroke is high, thus highlighting the necessity for optimal management of minor stroke patients.

The current mainstay in the management of minor stroke and secondary prevention is dual antiplatelet therapy (DAPT).^34^ Several studies have explored comparing the efficacy of thrombolytic therapy compared to non‐thrombolytic therapy; studies like the Antiplatelet vs R‐tPA for Acute Mild Ischemic Stroke (ARAMIS) trial have found that DAPT is non‐inferior to tPA.^35^, ^36^ Similar studies such as the Tenecteplase versus Standard of Care for Minor Ischemic Stroke with Proven Occlusion (TEMPO‐2) trial comparing TNK with DAPT showed uncertain benefit and potential disadvantages.^37^ The Potential of rtPA for Ischemic Strokes with Mild Symptoms (PRISMS) trial comparing tPA and aspirin similarly found no benefit.^38^ Thus, although studies show neutral or mixed outcomes using thrombolytics in minor strokes, certain subpopulations, such as young patients, as demonstrated in our analysis, may potentially benefit from this intervention. Future studies may similarly explore factors that may be associated with variation in therapeutic benefit from interventions within the minor stroke subpopulation.

Limitations to this study include factors inherent to the retrospective design, including confounding and selection bias. This study was conducted at a single site; further multicenter analyses will expand the generalizability of the results. Guidelines for practices regarding interventions for minor stroke have varied within the timeframe included within this analysis, which could confound these results; consequently, future external validation with additional data, especially from a wide range of sites that are representative of the diversity in approaches to managing minor ischemic stroke, and the changing guidelines over time, is required.

In conclusion, there is an age‐dependent effect of thrombolytic treatment on the likelihood of clinical symptom improvement in minor stroke patients. Thrombolytic treatment, particularly TNK, was associated with increased likelihood of clinical improvement in younger populations. Although recent studies show mixed benefits in the administration of IVT and other interventions of minor stroke, future studies may explore the benefit of thrombolytic administration in younger individuals.

## AUTHOR CONTRIBUTIONS

Tarun Thomas and Manisha Koneru conceptualized the study. Tarun Thomas, Michael J. Dubinski, Omnea Elgendy, Sonali Vij, Lucas P. Garfinkel, Karan Patel, Jesse M. Thon, Pratit D. Patel, Hamza A. Shaikh, Jane Khalife, Hermann C. Schumacher, and Khalid A. Hanafy collected the data. Manisha Koneru performed the data analysis. Pratit D. Patel, Hamza A. Shaikh, Jane Khalife, Hermann C. Schumacher, Khalid A. Hanafy, Tudor G. Jovin, and Manisha Koneru supervised the study. Tarun Thomas and Manisha Koneru wrote the original draft of the manuscript. Tarun Thomas, Michael J. Dubinski, Omnea Elgendy, Sonali Vij, Lucas P. Garfinkel, Karan Patel, Jesse M. Thon, Pratit D. Patel, Hamza A. Shaikh, Jane Khalife, Hermann C. Schumacher, Khalid A. Hanafy, Tudor G. Jovin, and Manisha Koneru edited and revised the manuscript. All authors read and approved the final version of this manuscript.

## CONFLICT OF INTEREST STATEMENT

The authors declare no conflicts of interest.

## ETHICS STATEMENT

This retrospective study was approved by the Cooper Hospital Institutional Review Board (#IRB 20‐371). The requirement for informed consent was waived because only deidentified data were analyzed.

## Data Availability

Deidentified data may be made available upon reasonable request to the corresponding author.
